# Lipid-Induced Endothelial Dysfunction: Pro-Atherogenic Properties of Multinucleated Variant Endothelial Cells

**DOI:** 10.3390/ijms27135728

**Published:** 2026-06-25

**Authors:** Vadim Cherednichenko, Diana Kiseleva, Ulyana Khovantseva, Rustam Ziganshin, Denis Fotin, Elena Zakharova, Olga Dymova, Alexander M. Markin

**Affiliations:** 1 Petrovsky National Research Center of Surgery, 119435 Moscow, Russia; 2 Department of Biophysics, Faculty of Biology, Lomonosov Moscow State University, 119991 Moscow, Russia; 3 Department of Biology and Genetics, Petrovsky Medical University, 119435 Moscow, Russia; 4 Shemyakin-Ovchinnikov Institute of Bioorganic Chemistry, Russian Academy of Sciences, 117997 Moscow, Russia; ziganshin@mail.ru; 5 Medical and Biological Faculty, Pirogov Russian National Research Medical University, 117997 Moscow, Russia; 6 Department of Histology, Petrovsky Medical University, 119435 Moscow, Russia

**Keywords:** endothelial dysfunction, multinucleated variant endothelial cells, low-density lipoproteins, atherosclerosis, endothelial barrier function, cholesterol accumulation, transendothelial macrophage migration, interleukin-6, interleukin-8, secretome proteomics

## Abstract

Endothelial dysfunction is an early event in the development of cardiovascular diseases and is characterized by impaired barrier function, inflammatory activation of endothelial cells (ECs), and alterations in lipid metabolism. In addition to typical (mononuclear) endothelial cells (TECs), multinucleated variant endothelial cells (MVECs) are present within the vascular wall; however, their functional role remains poorly understood. The aim of the present study was to investigate the molecular and functional characteristics of MVECs and their potential contribution to the development of endothelial dysfunction. Primary human umbilical vein endothelial cells (HUVECs) were used, and multinucleated cells were generated by polyethylene glycol-induced fusion. Cells were incubated under control conditions or exposed to low-density lipoproteins (LDL; 100 µg/mL, 24 h). A comprehensive analysis was performed, including transcriptomic and proteomic (secretome) profiling using gene set enrichment analysis (GSEA), as well as functional assays assessing transendothelial LDL transport, intracellular cholesterol accumulation, macrophage migration, and the expression and secretion of pro-inflammatory cytokines (IL-6, IL-8). MVECs exhibited pronounced differences compared to TECs. GSEA revealed reduced enrichment of pathways related to canonical nuclear factor kappa B (NF-κB) signaling and negative regulation of NF-κB transcription factor activity, actin cytoskeleton organization, focal adhesion assembly, basement membrane organization, and vesicle-mediated transport in MVECs relative to TECs, indicating impaired cytoskeletal integrity, altered cell–matrix interactions, dysregulated inflammatory signaling, and reduced vesicular trafficking activity. Functionally, MVECs demonstrated an increased capacity for cholesterol accumulation and enhanced transendothelial migration of macrophages. Notably, transendothelial LDL transport across the MVEC monolayer was not increased, suggesting a predominance of intracellular lipid accumulation. MVECs also exhibited a pronounced pro-inflammatory phenotype, characterized by elevated expression and secretion of IL-6 and IL-8. Taken together, these findings indicate that MVECs represent a functionally altered endothelial phenotype with impaired barrier function, dysregulated lipid metabolism, and enhanced inflammatory activity. Local accumulation of MVECs within the vascular wall may contribute to the formation of pro-atherogenic regions and play a role in the initiation and progression of endothelial dysfunction.

## 1. Introduction

Endothelial dysfunction (ED) is an early event in the development of cardiovascular diseases, including atherosclerosis. It is characterized by impaired endothelial barrier function, alterations in vascular tone, and an enhanced inflammatory response. One of the major factors contributing to ED is exposure to low-density lipoproteins (LDL), which can induce endothelial cell activation [1].

The human endothelium is highly heterogeneous and comprises multiple cell populations that differ not only morphologically but also functionally. In addition to typical (mononuclear) endothelial cells (TECs), multinucleated variant endothelial cells (MVECs) have been described in the vascular endothelium. Their presence has been associated with pathological conditions of the vascular wall, including atherosclerosis, aneurysm formation, aging, and chronic inflammation [2,3,4]. MVECs are typically localized in small clusters within the endothelium [2]. These cells contain multiple nuclei (three or more), exhibit aneuploidy, and demonstrate increased uptake of LDL [5].

MVECs have been identified in several vascular tissues, including the human aorta [6,7] and renal allograft microvasculature [3]. Notably, their presence has been reported not only in established atherosclerotic lesions but also in histologically normal-appearing areas of the aortic wall in aged individuals [2], suggesting that MVEC accumulation may precede overt pathological remodeling and could represent an early marker of endothelial vulnerability.

Understanding the contribution of MVECs to disease progression is of particular interest. Given that atherosclerotic lesions develop in a focal manner, clusters of MVECs may act as local “initiators” of vascular injury.

However, isolation and long-term cultivation of MVECs from native endothelium remain technically challenging due to their low abundance in vivo. In addition, multinucleated and polyploid cells are known to exhibit reduced proliferative capacity and cell cycle arrest, limiting their expansion in culture [8]. Various approaches have been proposed to enrich MVEC populations in vitro, including treatment with meso-4,4′-(2,3-butanediyl) bis(2,6-piperazinedione) (ICRF-193), LDL, interleukin-4 (IL-4), polyethylene glycol (PEG) [9,10], hydrogen peroxide (H_2_O_2_), and linoleic acid hydroperoxide (LAHO) [7]. In the present study, a triple treatment with 50% PEG 6000 was used to generate a heterogeneous cell population enriched in MVECs, in which multinucleated cells occupied at least 50% of the monolayer surface, enabling the investigation of their contribution to endothelial functional alterations.

In previous studies, the EA.hy926 endothelial cell line was used to model MVECs. In the present work, primary HUVECs were employed to better approximate physiological conditions. Despite their venous origin, HUVECs are widely used as a model of endothelial function due to their retention of key endothelial characteristics and their suitability for studying general mechanisms of endothelial activation. Nevertheless, it should be noted that HUVECs do not fully recapitulate the properties of arterial endothelium, particularly those shaped by specific hemodynamic conditions [11].

Transendothelial transport of lipoproteins and migration of immune cells across the endothelium are two key processes underlying atherosclerotic plaque formation [12]. Under physiological conditions, the endothelium selectively regulates the passage of LDL into the subendothelial space, limiting its accumulation within the intima [13]. Disruption of this barrier leads to increased lipid deposition, oxidative modification, and activation of inflammatory cascades [14]. In addition, the endothelium controls the adhesion and migration of monocytes and macrophages, which subsequently differentiate into foam cells and sustain chronic inflammation within the vessel wall [15]. Therefore, assessment of these two processes provides a direct evaluation of the pro-atherogenic potential of the studied endothelial cell populations.

The aim of the present study was to investigate the molecular and functional characteristics of MVECs using transcriptomic and proteomic analyses, as well as functional assays, including evaluation of transendothelial LDL transport and immune cell migration.

## 2. Results

Cells in this study were analyzed under control conditions and after treatment with native low-density lipoproteins (LDL), which are commonly used to model atherogenic stimuli and the development of endothelial dysfunction. Although HUVECs are of venous origin, they remain one of the most widely used models for studying endothelial cell activation, inflammation, and barrier dysfunction [11,16]. It has been demonstrated that LDL can induce HUVEC activation, including the activation of NF-κB-dependent signaling pathways and the inflammatory response [17,18].

**Figure 1:**
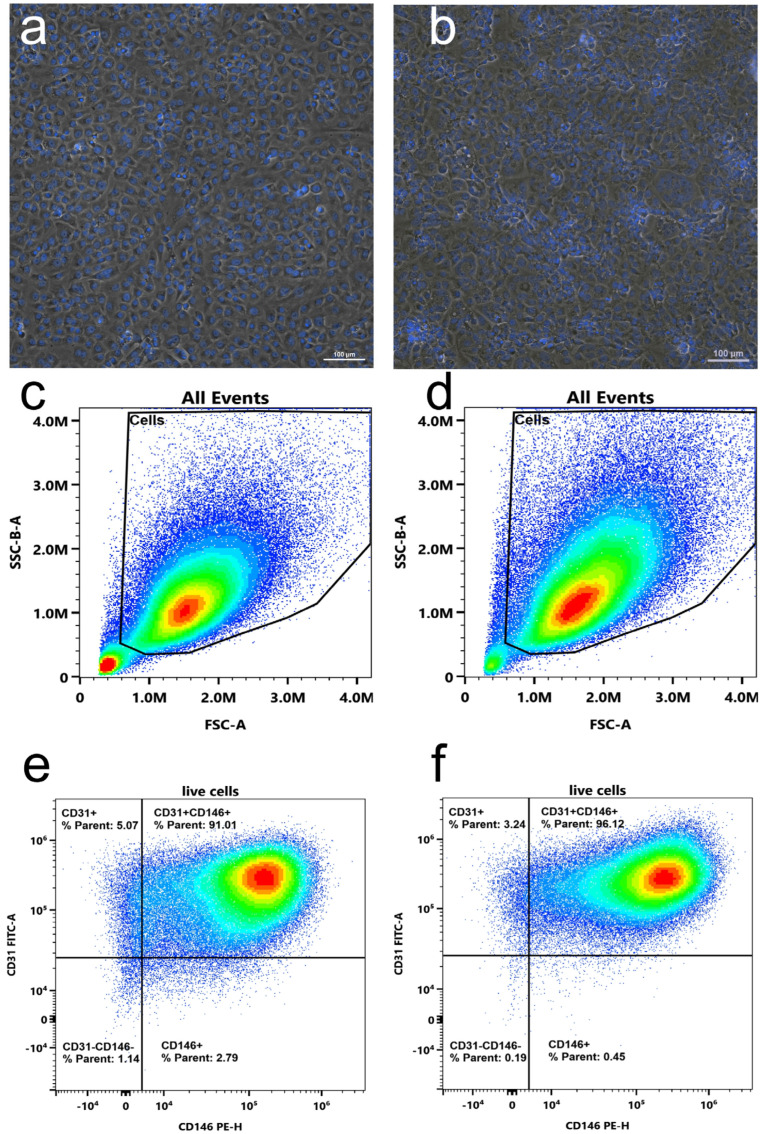
Morphological and phenotypic characterization of typical endothelial cells (TECs) and multinucleated variant endothelial cells (MVECs). (**a**,**b**) Fluorescence microscopy images of TEC (**a**) and MVEC (**b**) monolayers. Cell nuclei are stained with 4′,6-diamidino-2-phenylindole (DAPI) (blue); cell morphology is visualized by phase-contrast. Scale bar = 100 µm. MVECs exhibit a characteristic multinucleated morphology with enlarged cell bodies compared to TECs. (**c**,**d**) Flow cytometry scatter plots (FSC-A vs. SSC-B-A) showing the gating strategy for live cell populations in TECs (**c**) and MVECs (**d**). The “Cells” gate was applied to exclude debris and dead cells. The shift of the MVEC population toward higher SSC values reflects increased cell complexity and granularity associated with multinucleation. (**e**,**f**) Flow cytometry analysis of endothelial marker expression in TECs (**e**) and MVECs (**f**). Cells were stained with anti-CD31 (FITC) and anti-CD146 (PE). The CD31+CD146+ double-positive population, representing endothelial cells, constituted 91.01% and 96.12% of live cells in TECs and MVECs, respectively, confirming the endothelial identity of both cell populations. Colors indicate event density, ranging from low density (blue) to high density (red). Flow cytometry data are presented for n = 5 independent biological replicates. FSC-A and SSC-A differences between TECs and MVECs were assessed using Student’s independent samples *t*-test.

Morphological analysis confirmed successful induction of multinucleation following PEG treatment. TECs displayed a typical cobblestone monolayer morphology with uniform mononuclear cells (Figure 1a). MVECs exhibited enlarged, irregularly shaped cells with multiple nuclei visible within a single cell body (Figure 1b). Manual counting of 604 cells across 10 randomly selected regions of interest (ROIs) revealed that MVECs constituted 6.0% of the total cell population (36 of 604 cells), with a mean proportion of 6.1 ± 1.9% per ROI (range 2.6–8.6%).

Flow cytometry scatter analysis revealed that MVECs exhibited significantly higher forward scatter (FSC-A) values compared to TECs, reflecting increased cell size. The mean FSC-A was 1.894 ± 0.013 × 10^6^ in MVECs versus 1.726 ± 0.068 × 10^6^ in TECs (*p* < 0.001), corresponding to a 1.10-fold increase. Side scatter (SSC-A), reflecting intracellular complexity and granularity, showed a trend toward higher values in MVECs (mean SSC-A: 0.580 ± 0.021 × 10^6^ vs. 0.528 ± 0.061 × 10^6^ in TECs), however this difference did not reach statistical significance (*p* = 0.108). These data indicate that multinucleation is primarily associated with an increase in cell size rather than a proportional increase in intracellular granularity.

Flow cytometry analysis confirmed the endothelial identity of both cell populations. In TECs, 91.01% of live cells were CD31+CD146+ double-positive (Figure 1e). In MVECs, the proportion of CD31+CD146+ cells was 96.12% (Figure 1f). The proportion of CD31−CD146− double-negative cells was 1.14% in TECs and 0.19% in MVECs, indicating high purity of both populations and the absence of significant non-endothelial contamination.

To assess molecular differences between TECs and MVECs, transcriptomic and proteomic (secretome) data were analyzed using gene set enrichment analysis (GSEA). The analysis was focused on six pathways relevant to endothelial biology: canonical NF-κB signaling (GOBP_CANONICAL_NF_KAPPAB_SIGNAL_TRANSDUCTION), negative regulation of NF-κB transcription factor activity (GOBP_NEGATIVE_REGULATION_OF_NF_KAPPAB_TRANSCRIPTION_FACTOR_ACTIVITY), vesicle-mediated transport to the plasma membrane (GOBP_VESICLE_MEDIATED_TRANSPORT_TO_THE_PLASMA_MEMBRANE), regulation of actin filament organization (GOBP_REGULATION_OF_ACTIN_FILAMENT_ORGANIZATION), focal adhesion assembly (GOBP_FOCAL_ADHESION_ASSEMBLY), and basement membrane organization (GOBP_BASEMENT_MEMBRANE_ORGANIZATION). These pathways were selected based on their established relevance to endothelial barrier function, cytoskeletal integrity, lipid transport, and inflammatory signaling.

The proteomic and transcriptomic analyses were performed with n = 3 independent biological replicates. While this sample size is limited, it is consistent with published DIA-LC-MS-based secretome studies of primary endothelial cells, where biological variability is typically low and n = 3 replicates have been shown to provide sufficient statistical power for exploratory analyses [19]. Principal component analysis (PCA) of both transcriptomic and proteomic datasets confirmed that biological replicates clustered together within each condition, with clear separation between TEC and MVEC sample groups, supporting the reproducibility of the data (Figure S1). The complete list of identified secretome proteins and their differential abundance between TECs and MVECs is provided in Supplementary Table S1, and the leading edge genes for each significantly enriched gene set are listed in Supplementary Table S2. Accordingly, an false discovery rate (FDR) threshold of q < 0.25 was applied for GSEA, in accordance with recommendations for exploratory studies and the original methodology [20]. All proteomic findings should therefore be considered preliminary and are intended to generate hypotheses for subsequent validation.

**Figure 2:**
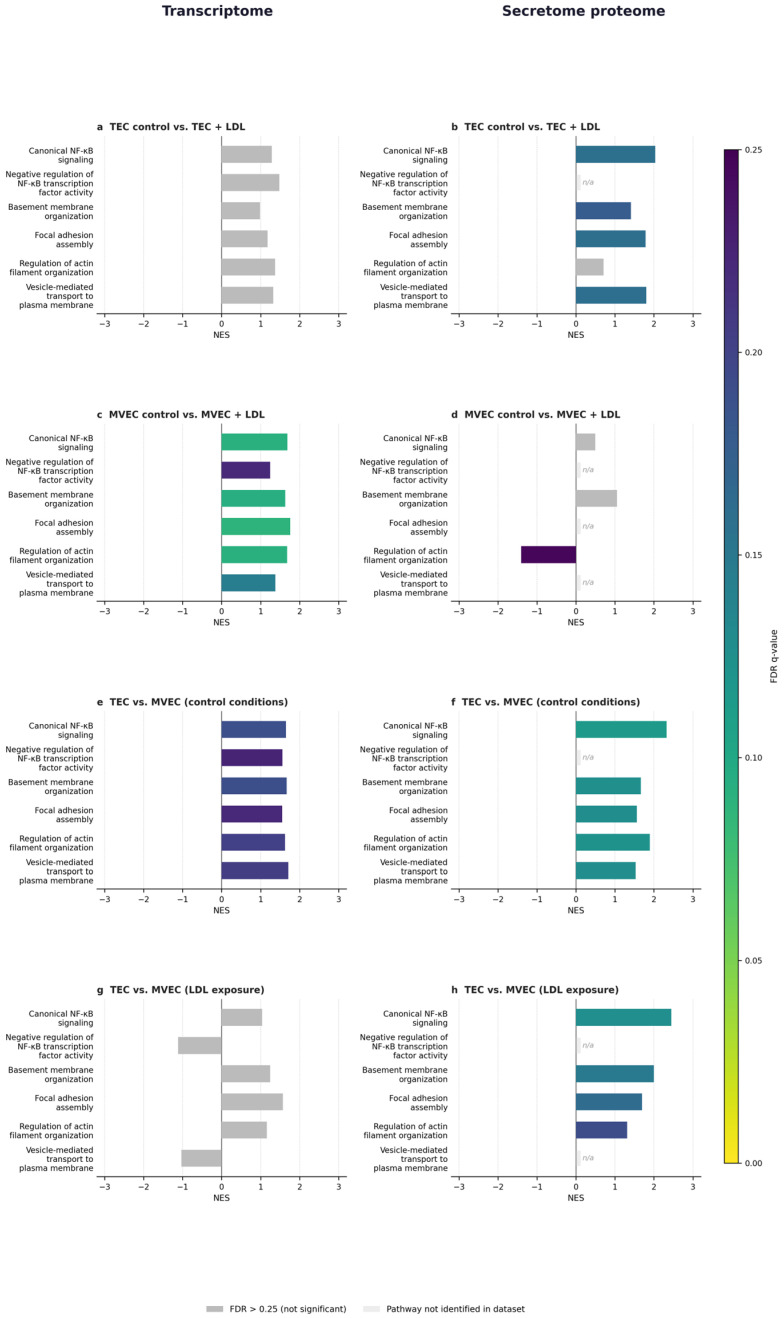
Gene set enrichment analysis (GSEA) of transcriptomic and proteomic (secretome) data in typical endothelial cells (TECs) and multinucleated variant endothelial cells (MVECs) under control conditions and following LDL exposure. Results are presented as normalized enrichment scores (NES). Positive NES values indicate enrichment in the first group of each comparison, whereas negative values indicate enrichment in the second group. Comparisons include: TEC control vs. TEC + LDL ((**a**) transcriptome, (**b**) secretome proteome); MVEC control vs. MVEC + LDL ((**c**) transcriptome, (**d**) secretome proteome); TEC vs. MVEC under control conditions ((**e**) transcriptome, (**f**) secretome proteome); and TEC vs. MVEC under LDL exposure ((**g**) transcriptome, (**h**) secretome proteome). The color scale represents the false discovery rate (FDR q-value), with lower values indicating higher statistical significance (n = 3). Gray bars indicate pathways with FDR > 0.25 (not significant); ‘n/a’ denotes pathways not identified in the dataset.

In TECs exposed to LDL, transcriptomic analysis did not reveal statistically significant enrichment (FDR < 0.25) for any of the six pathways, with normalized enrichment score (NES) values ranging from +1.29 to +1.37 and FDR values between 0.44 and 0.64, indicating a trend toward reduced pathway activity in the LDL-treated group that did not reach statistical significance (Figure 2a). In contrast, secretome proteomic analysis revealed significant enrichment of four pathways in TEC controls relative to LDL-treated TECs: canonical NF-κB signaling (NES = +2.04; FDR = 0.16), vesicle-mediated transport to the plasma membrane (NES = +1.80; FDR = 0.16), focal adhesion assembly (NES = +1.78; FDR = 0.16), and basement membrane organization (NES = +1.41; FDR = 0.18), indicating their reduced representation in the secretome following LDL exposure (Figure 2b).

When comparing MVECs under control conditions and after LDL exposure, transcriptomic analysis showed statistically significant enrichment for five of six pathways in the control group (FDR < 0.25), with positive NES values ranging from +1.38 to +1.84 (Figure 2c). The strongest transcriptomic signal among NF-κB-related pathways was observed for negative regulation of NF-κB transcription factor activity (NES = +1.25; FDR = 0.22) and regulation of actin filament organization (NES = +1.68; FDR = 0.09), indicating reduced activity of these pathways upon LDL treatment. Secretome proteomic analysis identified a single significantly enriched pathway—regulation of actin filament organization—but with a negative NES value (NES = −1.41; FDR = 0.25), indicating its enrichment in the LDL-treated group (Figure 2d). This discrepancy in direction between transcriptomic and proteomic data likely reflects the fact that actin cytoskeleton remodeling is a rapid posttranslational process that can occur independently of transcriptional regulation [21]. Additionally, as the proteomic analysis was performed on the secretome, increased representation of actin-associated proteins in conditioned medium may reflect active secretion or release from stress-activated cells rather than intracellular upregulation.

The comparison between TECs and MVECs under control conditions revealed the most consistent and comprehensive pattern of pathway enrichment across both omics layers (Figure 2e,f). All six analyzed pathways showed positive NES values in transcriptomic data, indicating enrichment in TECs, and all were statistically significant (FDR < 0.25), with NES values ranging from +1.47 to +1.71. At the secretome proteomic level, five of six pathways were also significantly enriched in TECs (FDR < 0.25), with NES values from +1.53 to +2.33. The only pathway absent from the proteomic dataset was negative regulation of NF-κB transcription factor activity, likely due to insufficient mass spectrometry coverage of the corresponding regulatory proteins, as low-abundance inhibitory proteins such as IκB family members are frequently underrepresented in secretome analyses.

Canonical NF-κB signaling was enriched in TECs at both the transcriptomic (NES = +1.65; FDR = 0.19) and proteomic (NES = +2.33; FDR = 0.12) levels. Concurrently, negative regulation of NF-κB transcription factor activity was enriched in TECs at the transcriptomic level (NES = +1.56; FDR = 0.225), indicating that TECs maintain active transcriptional suppression of NF-κB alongside its activation. In MVECs, reduced enrichment of negative regulatory components may contribute to a disinhibited inflammatory state, consistent with the elevated secretion of IL-6 and IL-8 observed in these cells.

Pathways related to cytoskeletal organization and cell–matrix interactions were consistently enriched in TECs in both omics layers. Regulation of actin filament organization was significantly enriched in TECs in both transcriptomic (NES = +1.63; FDR = 0.20) and proteomic (NES = +1.89; FDR = 0.12) analyses, suggesting a higher degree of cytoskeletal organization in TECs compared to MVECs. Similarly, focal adhesion assembly (transcriptome: NES = +1.55; FDR = 0.22; secretome: NES = +1.56; FDR = 0.13) and basement membrane organization (transcriptome: NES = +1.67; FDR = 0.19; secretome: NES = +1.67; FDR = 0.13) were enriched in TECs, indicating that MVECs exhibit altered cell–matrix adhesion and reduced extracellular matrix deposition.

The vesicle-mediated transport pathway (GOBP_VESICLE_MEDIATED_TRANSPORT_TO_THE_PLASMA_MEMBRANE) was significantly enriched in TECs at both the transcriptomic (NES = +1.71; FDR = 0.21) and proteomic (NES = +1.53; FDR = 0.13) levels, indicating higher activity of vesicular trafficking in TECs relative to MVECs. Reduced enrichment of this pathway in MVECs may reflect impaired intracellular lipid trafficking and contribute to the enhanced cholesterol accumulation observed in these cells.

Under LDL exposure, the comparison of TECs and MVECs did not reveal statistically significant differences at the transcriptomic level for most pathways. However, proteomic analysis demonstrated that differences in pathway enrichment between the two cell types persisted and were statistically significant for canonical NF-κB signaling (NES = +2.45; FDR = 0.13), regulation of actin filament organization (NES = +1.31; FDR = 0.19), focal adhesion assembly (NES = +1.69; FDR = 0.16), and basement membrane organization (NES = +2.00; FDR = 0.15), all enriched in TECs (Figure 2h). This suggests that functional differences between TECs and MVECs under lipid loading are primarily manifested at the secretome level, potentially reflecting post-translational regulation and differences in secretory activity.

**Figure 3:**
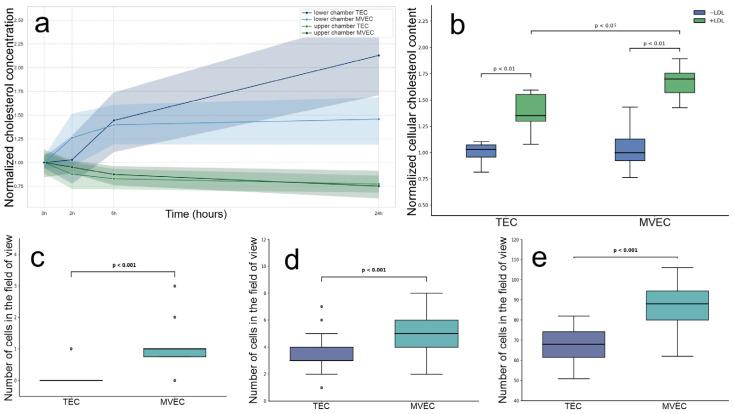
Functional analysis of transendothelial transport, cholesterol accumulation, and macrophage migration in typical endothelial cells (TECs) (HUVECs) and multinucleated variant endothelial cells (MVECs). (**a**) Time course of transendothelial LDL transport, presented as normalized cholesterol concentration in the lower chamber and upper chamber for TECs and MVECs. Data are presented as mean (thin, darker lines) ± SD (wide, lighter lines) (n = 8 independent biological replicates). (**b**) Normalized intracellular cholesterol content in TECs and MVECs under control conditions (−LDL) and following LDL treatment (+LDL). Data are presented as box plots showing the median, interquartile range, and full range (n = 12 independent biological replicates). (**c**–**e**) Transendothelial migration of M1-polarized macrophages across TEC and MVEC monolayers at 2, 5, and 24 h, respectively. Migrated cells in the lower chamber were counted in eight randomly selected fields of view per insert using an inverted phase-contrast microscope (magnification ×20). Data are presented as box plots showing the median and interquartile range (n = 8 independent biological replicates).

To assess the functional relevance of these findings, transendothelial transport (TET) of LDL was evaluated. At 2 h, no statistically significant differences between groups were observed. After 5 h, cholesterol concentration in the lower chamber increased in both groups and continued to rise up to 24 h (Figure 3a). At 24 h, cholesterol levels in the lower chamber were significantly higher in TECs compared to MVECs (*p* < 0.05), whereas cholesterol levels in the upper chamber (insert) decreased similarly in both groups.

Differences in LDL transport rates became evident after 24 h. Cholesterol concentration in the lower chamber was higher in TECs compared to MVECs, whereas cholesterol levels in the upper chambers decreased at a similar rate for both cell types. These findings suggest differences in LDL processing, with MVECs exhibiting a greater capacity for intracellular cholesterol accumulation. Consistently, MVECs accumulated LDL-derived cholesterol 19% more efficiently than TECs (*p* < 0.05) (Figure 3b).

Analysis of macrophage migration across endothelial monolayers revealed that no migration occurred within 24 h when monocytes were treated with GM-CSF. In contrast, M1-polarized macrophages demonstrated significantly higher migration rates across MVEC monolayers compared to TECs at 2, 5, and 24 h (*p* < 0.001) (Figure 3c–e).

Given that increased expression of IL-6 and IL-8 may promote monocyte recruitment and transendothelial migration, we next assessed cytokine expression and secretion using quantitative real-time PCR and ELISA.

**Figure 4:**
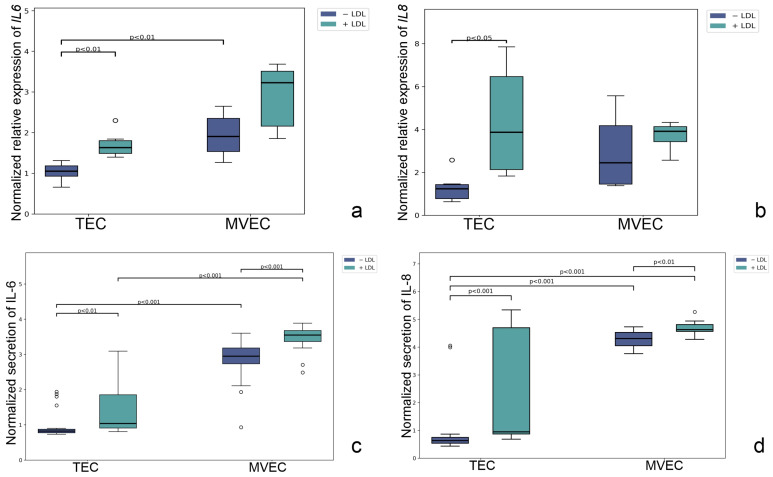
Expression and secretion of pro-inflammatory cytokines IL-6 and IL-8 in typical endothelial cells (TECs) and multinucleated variant endothelial cells (MVECs). (**a**) Relative mRNA expression of *IL6* measured by quantitative real-time PCR in TECs and MVECs under control conditions (−LDL) and after LDL treatment (+LDL). (**b**) Relative mRNA expression of *IL8*. (**c**,**d**) Secretion levels of IL-6 and IL-8 measured by ELISA in culture supernatants of TECs and MVECs under control conditions and following LDL exposure. Data are presented as box plots showing the median and interquartile range (n ≥ 6 independent biological replicates). Statistical significance was assessed using the Kruskal–Wallis test followed by Conover’s post hoc test. Differences were considered statistically significant at *p* < 0.05.

Basal *IL6* expression in MVECs was 1.9-fold higher than in TECs (*p* < 0.01). In addition, LDL treatment increased *IL6* expression in TECs by 1.7-fold (*p* < 0.01) (Figure 4a).

For *IL8*, a statistically significant change in expression was observed only when comparing TECs before and after LDL treatment, with a 3.5-fold increase in *IL8* expression (*p* < 0.05) (Figure 4b).

In contrast, differences at the level of secretion were more pronounced. Both TECs and MVECs exhibited increased secretion of IL-6 and IL-8 following LDL exposure. Moreover, cytokine secretion was significantly higher in MVECs compared to TECs, not only under basal conditions but also after LDL treatment.

**Figure 5:**
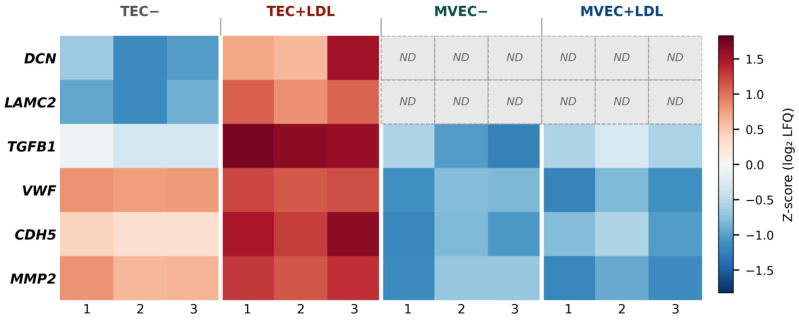
Secretome proteomic profile of endothelial marker proteins in typical endothelial cells (TEC) and multinucleated variant endothelial cells (MVEC) under control conditions and LDL exposure. Heatmap shows row-wise z-scores of log_2_-transformed label-free quantification (LFQ) intensities for 6 candidate marker proteins. Each column represents an independent biological replicate (n = 3 per group). Red indicates relatively higher abundance; blue indicates lower abundance. ND (not detected) denotes proteins with LFQ = 0 in all replicates of the respective group, shown as grey cells with dashed borders. Proteins were selected based on consistent absence (DCN, LAMC2) or marked reduction (VWF, CDH5, MMP2, TGFB1) in MVEC compared to TEC across both conditions. TEC−, typical endothelial cells without LDL treatment; TEC+LDL, typical endothelial cells treated with LDL; MVEC−, multinucleated variant endothelial cells without LDL; MVEC+LDL, multinucleated variant endothelial cells treated with LDL. Numbers 1–3 denote individual biological replicates.

To identify proteins that could potentially distinguish MVECs from TECs at the secretome proteome level, mass spectrometry-based analysis was performed. A total of 2240 proteins were identified in the secretome by mass spectrometry analysis. From this dataset, candidate proteins were selected based on three criteria: (1) statistically significant difference between TECs and MVECs under both experimental conditions (control and LDL-treated); (2) established functional relevance to endothelial biology, vascular homeostasis, or extracellular matrix organization; and (3) consistent direction of change (reduction or absence in MVECs relative to TECs) across both conditions. Applying these criteria, six candidate proteins were identified: decorin (DCN), laminin subunit gamma-2 (LAMC2), transforming growth factor beta-1 (TGFB1), von Willebrand factor (VWF), VE-cadherin (CDH5), and matrix metalloproteinase-2 (MMP2). Notably, several of these proteins are detectable in human blood and are already used or investigated as circulating biomarkers of vascular injury and endothelial dysfunction: VWF is a well-established plasma marker of endothelial activation [22], soluble CDH5 has been proposed as an indicator of endothelial barrier disruption [23], MMP2 circulates in plasma and is associated with arterial remodeling and atherosclerosis [24], and TGFB1 is measurable in serum and linked to vascular inflammation and fibrosis [25]. This suggests that the identified secretome alterations in MVECs may have potential relevance beyond the in vitro setting. The relative abundance of these proteins across all experimental groups is presented in Figure 5.

Decorin (DCN) and laminin subunit gamma-2 (LAMC2), which were detected in the TEC secretome and showed increased abundance upon LDL treatment, were not detected in MVECs under either control conditions or following LDL exposure.

For TGFB1, VWF, CDH5, and MMP2, the differences were quantitative. All four proteins were detected in both TECs and MVECs; however, their levels were substantially lower in MVECs. Under control conditions, VWF levels in the TEC secretome were 8.1-fold higher than in MVECs (*p* < 0.001), MMP2 3.9-fold higher (*p* < 0.001), CDH5 2.5-fold higher (*p* < 0.001), and TGFB1 1.4-fold higher (*p* < 0.05).

Following LDL exposure, these differences became more pronounced: VWF levels were 15.2-fold higher in TECs than in MVECs (*p* < 0.001), MMP2 8.0-fold higher (*p* < 0.001), CDH5 4.6-fold higher (*p* < 0.001), and TGFB1 2.8-fold higher (*p* < 0.001).

In TECs, LDL treatment significantly increased the levels of all four proteins: VWF by 1.6-fold (*p* < 0.001), CDH5 by 2.2-fold (*p* < 0.001), MMP2 by 1.7-fold (*p* < 0.01), and TGFB1 by 2.5-fold (*p* < 0.001). In contrast, no comparable response was observed in MVECs.

Statistical analysis was performed using one-way ANOVA with pairwise comparisons. Given the limited sample size (n = 3), these findings should be considered preliminary.

## 3. Discussion

The findings obtained in the present study provide new insights into the functional properties of MVECs and their potential role in the development of endothelial dysfunction. However, the interpretation of these results requires consideration of several limitations.

Human umbilical vein endothelial cells (HUVECs), which are of venous origin, were used as the experimental model. This model does not fully recapitulate the properties of arterial endothelium, where MVECs are more commonly observed. Nevertheless, HUVECs are widely used to study mechanisms of endothelial activation and inflammation, as key molecular processes underlying endothelial dysfunction, including activation of NF-κB-dependent signaling pathways and responses to lipid exposure, are conserved across different endothelial cell types [26,27,28,29,30]. In this study, MVECs were generated using a polyethylene glycol (PEG)-based approach, which is commonly employed to reproducibly induce multinucleation. However, PEG treatment may induce transient alterations in cell phenotype [9,10,31,32]. To minimize these effects, endothelial cells were cultured for at least 24 h in complete growth medium supplemented with FBS following the final PEG treatment, with morphological recovery and monolayer confluence visually monitored prior to downstream analyses. Despite these limitations, the model allowed for the identification of functional differences between TECs and MVECs and enabled assessment of the contribution of multinucleation to endothelial dysfunction.

The proportion of MVECs in the PEG-treated monolayer constituted 6.0% of the total cell population (mean 6.1 ± 1.9% per ROI), which is consistent with the reported abundance of MVECs in the vascular endothelium in vivo. Primary cultures from human aortas have shown that variant endothelial cells are interspersed throughout the monolayer alongside the typical cell type, and their ratio correlates with the severity of atherosclerosis. In human aortas, MVECs exist individually or in groups, surrounded by the majority of typical endothelial cells, and their number increases with atherosclerosis grade and age. Notably, MVECs are generally present not only in atherosclerotic aorta but also in non-atherosclerotic aortic tissue, suggesting that a baseline level of multinucleated cells is a physiological feature of the endothelium [2,5,7]. The observed proportion of 6% in the present model is therefore within the range reported in pathologically relevant endothelial preparations [3,6] and confirms successful enrichment of the MVEC population following PEG-induced fusion. This is further supported by the flow cytometry data, which demonstrated that both TEC and MVEC populations retained high expression of endothelial markers CD31 and CD146 (91.0% and 96.1% CD31+CD146+ cells, respectively), confirming the endothelial identity of the model and excluding significant non-endothelial contamination.

Formal quantitative assessment of cell viability following PEG-induced fusion and LDL exposure was not performed in the present study. While monolayer integrity and cell morphology were confirmed by phase-contrast microscopy throughout all experiments, and trypan blue exclusion confirmed > 95% viability under routine culture conditions, low-level cell death undetectable by morphological assessment cannot be excluded. Future studies should include quantitative viability assays (such as MTT or annexin V/propidium iodide flow cytometry) at multiple time points to formally characterize the viability profile of this primary HUVEC-based MVEC model independently of previous EA.hy926-based data.

The results demonstrate that MVECs exhibit distinct functional characteristics compared to TECs. In addition to alterations in cytoskeletal organization, cell–matrix adhesion, and vesicle-mediated transport, MVECs display a pronounced pro-inflammatory phenotype, characterized by increased expression and secretion of IL-6 and IL-8. GSEA revealed reduced enrichment of pathways related to canonical NF-κB signaling, actin filament organization, focal adhesion assembly, basement membrane organization, and vesicle-mediated transport in MVECs relative to TECs. These differences were most consistent in the direct comparison of TECs and MVECs under control conditions, where concordant enrichment in TECs was observed at both transcriptomic and secretome proteomic levels. The apparent discrepancy between reduced enrichment of the canonical NF-κB signaling pathway at the gene set level and elevated secretion of individual pro-inflammatory cytokines (IL-6, IL-8) in MVECs may be explained by the fact that enrichment analysis reflects coordinated changes across large gene or protein sets—including cytokines, receptors, inhibitors, and feedback regulators [20]—whereas alterations in individual cytokines may reflect specific phases of the inflammatory response [33]. A mechanistically important finding is the reduced enrichment of the pathway of negative regulation of NF-κB transcription factor activity (GOBP_NEGATIVE_REGULATION_OF_NF_KAPPAB_TRANSCRIPTION_FACTOR_ACTIVITY) in MVECs at the transcriptomic level [4]. This pathway was significantly enriched in TECs relative to MVECs under control conditions (NES = +1.56; FDR = 0.225) and showed reduced representation in MVECs upon LDL exposure (NES = +1.25; FDR = 0.221 in MVEC control vs. MVEC+LDL comparison). In TECs, both canonical NF-κB signaling and its negative regulators are co-enriched, suggesting a balanced regulatory state. In MVECs, reduced transcriptional representation of negative regulatory components—including members of the IκB family and other NF-κB inhibitors—may result in tonic disinhibition of this pathway, providing a mechanistic basis for the elevated baseline secretion of IL-6 and IL-8 observed in these cells. This pathway was not detected in the secretome proteome, likely reflecting insufficient mass spectrometry coverage of low-abundance inhibitory proteins, which are known to be underrepresented in secretome analyses. Notably, this regulatory mechanism operates primarily at the transcriptional level and does not require elevated expression of individual cytokine genes to produce a functional pro-inflammatory output, which is consistent with the observed pattern of gene expression data.

These findings partially differ from those reported in our previous EA.hy926-based study, where elevated NFKB1 mRNA expression was observed in MVECs at the transcript level. This apparent discrepancy may reflect differences between immortalised and primary cell models in the regulation of NF-κB signaling. In EA.hy926 cells, which carry chromosomal aberrations characteristic of their A549 carcinoma origin, basal NF-κB activity may be constitutively elevated. In primary HUVECs, the regulatory landscape may more faithfully reflect physiological endothelial NF-κB control, where the observed reduction in negative regulatory components rather than overt pathway activation may represent the dominant mechanism driving the pro-inflammatory phenotype. We acknowledge that direct confirmation of NF-κB activity (for example by nuclear translocation assay, NF-κB reporter assay, or phospho-p65 immunostaining) was not performed in the present study and represents an important direction for future work.

Importantly, increased secretion of IL-6 and IL-8 may exert effects not only on immune cells but also on neighboring endothelial cells, thereby creating a local microenvironment that sustains endothelial activation [34,35,36].

Elevated levels of IL-6 and IL-8 in MVECs are functionally significant. Increased expression and secretion of these cytokines in multinucleated variant endothelial cells was previously demonstrated in the EA.hy926 cell line [4]; the present study confirms and extends these findings in primary HUVECs, providing additional evidence that this pro-inflammatory phenotype is a consistent feature of MVECs independent of the cell model used. IL-6 contributes to endothelial activation and enhances the expression of adhesion molecules [34,37], whereas IL-8 is a key chemoattractant regulating leukocyte migration across the endothelial layer [38,39], modulates barrier function [40], and inhibits apoptosis [41].

Given that MVECs are localized in clusters, their presence may lead to the formation of focal regions within the endothelium characterized by increased permeability and a pro-inflammatory microenvironment. This is particularly relevant, as functionally altered local regions may promote the development of vascular pathology. Since atherosclerosis develops in a spatially heterogeneous manner within the vessel wall [1], such altered endothelial areas may contribute to the initiation and/or progression of lesions at specific sites. Thus, the local accumulation of MVECs may represent an early marker of pathological processes within the endothelium.

The results of the transendothelial transport (TET) assay did not confirm the previously proposed hypothesis of enhanced LDL transport across endothelial monolayers containing MVECs [5]. However, MVECs exhibited a greater capacity for cholesterol accumulation compared to TECs, consistent with previous observations demonstrating enhanced lipid accumulation in MVECs relative to mononuclear endothelial cells in primary endothelial cultures [42], in the EA.hy926 endothelial cell line [4], and in vascular tissue samples containing MVECs [2]. The present study thus confirms this phenomenon in primary HUVECs and supports the conclusion that increased intracellular cholesterol accumulation is a consistent and reproducible feature of the MVEC phenotype across different experimental models. These findings suggest that MVECs may contribute not to lipoprotein transport, but rather to their accumulation within the endothelium. Excessive intracellular cholesterol accumulation may, in turn, alter endothelial cell metabolism, affecting the expression of adhesion molecules, impairing barrier function, and modifying endothelial responses to hemodynamic forces [43]. In the present study, we also observed reduced representation of pathways associated with actin cytoskeleton organization, focal adhesion assembly, basement membrane organization, and canonical NF-κB signaling in endothelial cells exposed to LDL, primarily at the secretome proteomic level. These changes were most pronounced in TECs, where LDL exposure was associated with significant reduction in enrichment of canonical NF-κB signaling, vesicle-mediated transport, focal adhesion assembly, and basement membrane organization pathways in the secretome, suggesting that LDL induces a broad reorganization of secretory and structural programs even in morphologically normal endothelial cells.

In addition, LDL may undergo intracellular modification, giving rise to more atherogenic forms, including oxidized LDL (oxLDL) [44]. Intracellular oxLDL has been shown to induce endothelial nitric oxide synthase (eNOS) uncoupling, thereby reducing nitric oxide (NO) production and impairing vasodilation [45]. Thus, the increased capacity of MVECs for cholesterol accumulation may be accompanied by intracellular LDL modification, further contributing to the development of endothelial dysfunction.

Alterations in pathways related to vesicle-mediated transport suggest a potential reorganization of intracellular trafficking mechanisms in MVECs. Vesicular transport regulates not only protein secretion but also intracellular lipid distribution [46,47], including the uptake, transport, and sorting of LDL [46,48]. Changes in the representation of components of vesicular transport pathways may indicate impaired lipid trafficking and contribute to intracellular lipid accumulation. This interpretation is consistent with our findings, as MVECs exhibited reduced enrichment of vesicle-mediated transport pathways alongside increased LDL accumulation. Similar trends were observed under both control conditions and LDL exposure. Taken together, these results suggest that alterations in vesicular transport may link disrupted lipid metabolism, enhanced secretion of pro-inflammatory mediators, and impaired barrier function in MVECs. Nevertheless, although these data indicate an association between MVECs, vesicular transport dysregulation, lipid accumulation, and inflammatory activation, causal relationships between these processes require further investigation.

Reduced enrichment of focal adhesion assembly (GOBP_FOCAL_ADHESION_ASSEMBLY) and basement membrane organization (GOBP_BASEMENT_MEMBRANE_ORGANIZATION) in MVECs, observed consistently at both transcriptomic and secretome proteomic levels, provides additional evidence of impaired cell–matrix interactions in these cells. Focal adhesions serve as mechanosensory structures linking the actin cytoskeleton to the extracellular matrix, and their disruption is associated with increased endothelial permeability and impaired barrier function [49,50]. Reduced representation of basement membrane organization pathways in the MVEC secretome is directly corroborated by the absence of DCN and LAMC2 (key basement membrane components) in the MVEC secretome, as well as by markedly reduced levels of TGFB1, which plays a central role in extracellular matrix remodeling and basement membrane maintenance [51,52].

Secretome proteomic analysis revealed characteristic alterations in the protein profile of MVECs that may be relevant for identifying this cellular phenotype. Decorin (DCN) and laminin subunit gamma-2 (LAMC2), key components of the extracellular matrix (ECM) and basement membrane, were not detected in the MVEC secretome. DCN is involved in collagen matrix organization and exhibits antiproliferative properties [53], whereas LAMC2 contributes to basement membrane integrity and cell–matrix adhesion [51]. Reduced levels of these proteins in MVECs may indicate impaired cell–matrix interactions. In combination with alterations in junction-associated proteins, these findings further support the hypothesis of reduced barrier function in MVECs. Decreased secretion of ECM and basement membrane components may reflect a shift of MVECs toward a less stable phenotype characterized by increased permeability.

Levels of von Willebrand factor (VWF), VE-cadherin (CDH5), transforming growth factor beta-1 (TGFB1), and matrix metalloproteinase-2 (MMP2) were also reduced in MVECs compared to TECs. Notably, all four proteins exhibited a pronounced increase in secretion in TECs upon LDL exposure, whereas this response was markedly attenuated in MVECs. This suggests that MVECs are characterized not only by an altered basal secretome but also by a reduced capacity for adaptive remodeling of their secretory activity in response to an atherogenic stimulus. These changes in the MVEC secretome reflect a reorganization of cell–matrix interactions and altered regulation of the extracellular matrix, consistent with reduced junctional integrity as evidenced by decreased CDH5 secretion and reduced enrichment of focal adhesion assembly and basement membrane organization pathways [51]. Collectively, these features may contribute to increased endothelial permeability, enhanced inflammatory responses, and, ultimately, progression of pathological processes associated with endothelial dysfunction [1,54,55]. The observed secretome alterations define a characteristic proteomic profile of MVECs that may potentially be used for their identification. However, given the limited sample size (n = 3), these findings should be considered preliminary and require validation in independent biological models. Future studies should aim to validate the identified secretome alterations—particularly the reduced levels of VWF, CDH5, MMP2, TGFB1, DCN, and LAMC2—using ELISA or Western blotting in a larger number of biological replicates, and in additional endothelial cell models, including human arterial endothelial cells or ex vivo vascular tissue samples.

Overall, the results indicate that MVECs exhibit a distinct functional phenotype characterized by dysregulated NF-κB inflammatory signaling, impaired cytoskeletal organization, altered cell–matrix adhesion, reduced basement membrane deposition, dysregulated vesicular transport, and enhanced cholesterol accumulation, accompanied by increased secretion of pro-inflammatory cytokines IL-6 and IL-8. The convergence of transcriptomic and secretome proteomic data across multiple pathways—most consistently in the direct TEC vs. MVEC comparison—supports the conclusion that these alterations reflect stable phenotypic differences associated with multinucleation rather than transient responses to experimental conditions. A mechanistically coherent interpretation emerges from the combination of reduced negative regulation of NF-κB, impaired cytoskeletal and matrix organization, and dysregulated vesicular transport: together, these changes may create a self-sustaining pro-atherogenic microenvironment characterized by increased permeability, enhanced inflammatory signaling, and impaired adaptive responses to lipid loading. Local accumulation of MVECs within the vascular wall may therefore contribute to the formation of focal pro-atherogenic regions and play a role in the initiation and progression of endothelial dysfunction.

## 4. Materials and Methods

### 4.1. Experimental Design

Both cell types (TECs and MVECs) were derived from the same HUVEC source and used in parallel experiments. TECs were untreated HUVECs serving as the mononuclear control population, whereas MVECs were generated from HUVECs by PEG-induced fusion (see Section 4.3). After generation of MVECs, both cell types were seeded at equal densities and cultured to confluence under identical conditions. Cells were routinely maintained in Endo-LV Growth medium supplemented with 2% FBS. For all experimental incubations, the maintenance medium was replaced with serum-free medium containing either LDL (100 µg/mL) or no additives (control) for 24 h prior to sample collection. Incubation was carried out for 24 h at 37 °C in a humidified atmosphere with 5% CO_2_.

Following incubation, cells and culture supernatants were collected for analysis. The experimental workflow comprised 7 major parts:

1. transcriptomic analysis;
2. proteomic (secretome) analysis
3. assessment of transendothelial LDL transport;
4. analysis of intracellular cholesterol accumulation;
5. evaluation of transendothelial macrophage migration;
6. assessment of the expression and secretion of pro-inflammatory cytokines;
7. flow cytometry analysis of endothelial marker expression.

**Figure 6:**
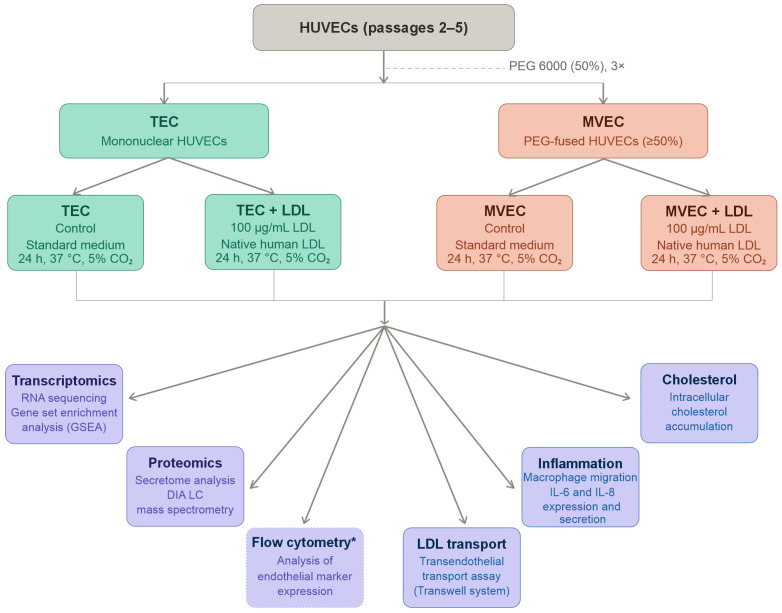
Experimental design. Primary human umbilical vein endothelial cells (HUVECs, passages 2–5) were used to generate two cell populations: typical endothelial cells (TECs, untreated mononuclear HUVECs) and multinucleated variant endothelial cells (MVECs, obtained by three consecutive treatments with 50% polyethylene glycol (PEG) 6000). Both cell types were cultured to confluence and incubated for 24 h at 37 °C in 5% CO_2_ either in standard culture medium (control) or in medium supplemented with 100 µg/mL of native human low-density lipoprotein (LDL). Five analytical readouts were assessed: transcriptomic profiling by RNA sequencing with gene set enrichment analysis (GSEA); secretome proteomics by data-independent acquisition liquid chromatography–mass spectrometry (DIA-LC-MS); transendothelial LDL transport using a Transwell system; intracellular cholesterol accumulation; and inflammatory markers including transendothelial macrophage migration, expression and secretion of interleukin-6 (IL-6) and interleukin-8 (IL-8) and flow cytometry analysis of endothelial marker expression (CD31, CD146). * Flow cytometry was performed on cells under control conditions (without LDL exposure) to confirm the endothelial identity of both cell populations prior to functional assays.

The overall experimental design is summarized in Figure 6.

### 4.2. Cell Culture

HUVEC human umbilical vein endothelial cells, which were purchased from the Almazov National Research Medical Center of the Russian Ministry of Health, were used as the TECs model. The cells were kept in the Endo-LV Growth kit (Bioinnlabs, Rostov-on-Don, Russia) with low-serum (2% FBS, Biosera, Nuaille, France), which already contained all the necessary additives.

HUVECs from early passages (2–5) were used for experiments, which allowed minimizing the phenotypic changes associated with prolonged cultivation. The cells were cultured at 37 °C in a humid atmosphere with 5% CO_2_ content.

### 4.3. Induction of Multinucleated Endothelial Cells

To obtain MVECs, confluent monolayers of HUVEC were subjected to three consecutive treatments with a 50% polyethylene glycol (PEG) 6000 solution (PanEco, Moscow, Russia), which induced cell membrane fusion. This approach is widely used in classical cell fusion protocols [9,10].

Following PEG treatment, cells were carefully washed and allowed to recover for at least 24 h prior to functional assays. It has been previously shown that PEG-mediated fusion can induce transient stress responses and membrane remodeling [10,31,32]. Therefore, all downstream analyses in the present study were performed only after restoration of normal cell morphology and formation of a confluent monolayer. In the experimental model used by us, after treatment with polyethylene glycol, multinucleated endothelial cells constituted 6.1 ± 1.9% of the total cell number (36 of 604 cells counted across 10 randomly selected fields of view), while occupying a substantially larger proportion of the monolayer surface area (approximately 50%) due to their characteristically enlarged cell bodies. Quantitative analysis of the nuclei showed that the average number of nuclei was 6.5 ± 3.3 per cell.

### 4.4. LDL Isolation

LDL was isolated from human plasma by sequential density-gradient ultracentrifugation, as previously described [56,57,58]. Density solutions were prepared as follows: 1.006 g/cm^3^ NaCl/EDTA (11.42 g/L NaCl, 0.1 g/L EDTA in Milli-Q water) (PanEco, Moscow, Russia); 1.019 g/cm^3^ (16.5 g/L KBr (DIA-M, Moscow, Russia) in 1.006 g/cm^3^ NaCl/EDTA); 1.065 g/cm^3^ (77.1 g/L KBr in 1.006 g/cm^3^ NaCl/EDTA).

Crystalline KBr (0.5 g per 1 mL of plasma) was added to collected plasma and dissolved with gentle vortexing. The saline plasma was transferred into ultracentrifuge tubes to two-thirds of their final volume, and the remaining one-third was filled with the 1.019 g/cm^3^ KBr solution. Tubes were balanced (tolerance ± 0.01 g) and centrifuged at 40,000 rpm for 50 min at +4 °C using a pre-cooled rotor (Beckman L8-55M, Brea, CA, USA). After centrifugation, chylomicrons and HDL accumulated in the upper layer, whereas LDL remained at the phase separation boundary. The upper layer was removed, leaving 1–2 mm above the boundary. A 1.065 g/cm^3^ KBr solution was carefully layered on top (volume adjusted according to the amount of removed supernatant, ~4 mL for an initial 21 mL load). The tubes were rebalanced and centrifuged for 2 h 10 min at 40,000 rpm and +4 °C. After ultracentrifugation, LDL migrated into the upper fraction, forming a reddish-brown ring. The LDL fraction was collected by carefully pipetting at the ring level. The study was approved by the Local Ethics Committee of the National Research Center for Surgery Petrovsky (Approval No. 3 dated 17 March 2022).

### 4.5. Cell Seeding and LDL Treatment

TECs and MVECs were seeded into 24-well plates at a density of 5 × 10^5^ cells per 1 mL of DMEM/F12 medium supplemented with 10% FBS. After reaching 100% confluence, the culture medium was replaced with DMEM/F12 containing 100 µg/mL of purified LDL. Cells were incubated for 24 h at 37 °C in 5% CO_2_. Culture supernatants were collected and stored at −80 °C for subsequent ELISA and DIA-LC-MS Analysis.

The selected concentration (100 µg/mL) and incubation period (24 h) correspond to commonly used experimental conditions for modeling LDL-induced endothelial dysfunction in vitro and were chosen to ensure sufficient lipid accumulation while avoiding overt cytotoxicity [57,59]. LDL preparations were native (non-oxidized) human LDL obtained by sequential density-gradient ultracentrifugation.

### 4.6. Flow Cytometry

To assess the expression of endothelial markers CD31 and CD146, flow cytometry was performed using a Cytek Aurora™ CS spectral flow cytometer (Cytek Biosciences, Fremont, CA, USA). Following culture, cells were detached using 0.25% trypsin solution, neutralized with complete culture medium, and pelleted by centrifugation at 300× *g* for 5 min. The pellet was resuspended in phosphate-buffered saline (PBS).

Cells were incubated with fluorescently labeled antibodies against CD31 (FITC) and CD146 (PE), diluted according to the manufacturer’s recommendations, for 30 min at room temperature in the dark. Cells were then washed twice with PBS. Cell viability was assessed by addition of DAPI (1 ng/mL) immediately prior to acquisition; DAPI-positive events were excluded from analysis as non-viable cells.

Data acquisition was performed on the Cytek Aurora™ CS spectral flow cytometer using standard excitation and emission settings. A minimum of 10,000 events were recorded per sample. Spectral unmixing was performed using single-color controls in accordance with the manufacturer’s recommendations. Data analysis was carried out using SpectroFlo™ software, version 1.4.1 (Cytek Biosciences, Fremont, CA, USA).

Cell gating was performed based on forward and side scatter parameters (FSC/SSC) to exclude debris and non-cellular events. Live, DAPI-negative cells were gated for subsequent analysis of CD31 and CD146 expression. Marker expression levels were assessed by mean fluorescence intensity and the proportion of positive cells within the live cell gate.

### 4.7. RNA Isolation

Total RNA was isolated using the RUplus RNA extraction kit (Biolabmix, Novosibirsk, Russia) according to the manufacturer’s protocol. After removal of the culture medium, lysis buffer was added directly to the cells in the wells, followed by incubation for 10 min at room temperature. The lysate was collected and transferred to microtubes.

Lysates were centrifuged for 10 min at 10,000× *g*, and the supernatant was transferred to clean 1.5–2 mL tubes. To each sample, 400 μL of binding buffer was added, mixed by pipetting, and up to 800 μL of the mixture was applied onto the spin column. Columns were centrifuged for 30 s at 10,000× *g*, and the flow-through was discarded. If the total sample volume exceeded 800 μL, the remaining fraction was reapplied to the same column and centrifuged under the same conditions.

Columns were washed sequentially with 500 μL of wash buffer 1 and 500 μL of wash buffer 2 (supplemented with ethanol), each followed by centrifugation for 30 s at 10,000× *g*. Finally, columns were centrifuged for 3 min at 10,000× *g* to remove residual WB2.

Columns were placed into clean 1.5–2 mL tubes, and 60–200 μL of elution buffer was applied directly to the center of the membrane. After 1 min incubation at room temperature, RNA was eluted by centrifugation for 1 min at 10,000× *g*.

The concentration and purity of isolated RNA were measured using a NanoDrop spectrophotometer (Thermo Fisher Scientific, Waltham, MA, USA). RNA integrity was verified by agarose gel electrophoresis. Samples were stored at −80 °C until use for cDNA synthesis and transcriptome analysis.

### 4.8. cDNA Synthesis

First-strand cDNA was synthesized using the M-MuLV–RH reverse transcriptase kit (Biolabmix, Novosibirsk, Russia) according to the manufacturer’s instructions. Briefly, 0.1 ng–5 µg of total RNA was mixed with 1–3 µL of primers in nuclease-free water to a final volume of 12 µL. The mixture was heated at 70 °C for 2–3 min to denature secondary RNA structures and immediately chilled on ice.

A reaction mix containing 4 µL of 5× RT buffer, 1 µL of M-MuLV–RH reverse transcriptase (100 U/µL), and 3 µL of nuclease-free water was then added (final volume 20 µL). Reactions were incubated at 25 °C for 10 min followed by 60 min at 42 °C.

Reverse transcription was terminated by heating to 70 °C for 10 min. The resulting cDNA was either used immediately for qPCR or stored at −20 °C for short-term use and at −70 °C for long-term storage.

### 4.9. Quantitative Real-Time PCR (qPCR)

Quantitative PCR was performed using the commercial reaction mixture 5× qPCRmix-HS SYBR (Evrogen, Moscow, Russia) on a CFX96 Real-Time PCR Detection System (Bio-Rad, Hercules, CA, USA) according to the manufacturer’s instructions. Each reaction was carried out in a final volume of 25 µL, containing 2 µL of cDNA template, 5 µL of 5× qPCRmix-HS SYBR, 0.4 µM of each primer, and nuclease-free water.

Amplification conditions were as follows: initial denaturation at 95 °C for 3 min, followed by 40 cycles of 95 °C for 10 s and 60 °C for 30 s. Melt curve analysis was performed after amplification to verify the specificity of the PCR products.

Sequences of forward and reverse primers for IL-6 Forvard-AGACAGCCACTCACCTCTTCAG, Reverse-TTCTGCCAGTGCCTCTTTGCTG and IL-8 Forvard-GAGAGTGATTGAGAGTGGACCAC, Reverse-CACAACCCTCTGCACCCAGTTT. The primers were synthesized by Lumiprobe (Moscow, Russia), and their specificity was confirmed using the Primer-BLAST (NCBI) service (https://www.ncbi.nlm.nih.gov/tools/primer-blast/, which was accessed on 14 April 2025). Relative gene expression was calculated using the 2^−ΔΔCT^ method, using ACTB as an internal control.

### 4.10. Enzyme-Linked Immunosorbent Assay (ELISA)

The concentrations of secreted cytokines IL-6 and IL-8 were measured using the following commercial enzyme immunoassay kits (ELISA): Human IL-6 DuoSet ELISA and Human IL-8/CXCL8 DuoSet ELISA (R&DSystems Inc., Minneapolis, MN, USA).

The analysis was performed in 96-well plates with high protein binding capacity. The plates were pre-coated with a primary antibody diluted in PBS to the manufacturer’s recommended concentration, and incubated at room temperature overnight. After incubation, the wells were washed three times with PBS with 0.05% Tween-20 (PanEco, Moscow, Russia) (PBS-T) and blocked with 1% BSA (bovine serum albumin) (PanEco, Moscow, Russia) diluted in PBS at room temperature for 2 h.

100 µL of test samples or standards, diluted in 1% BSA/PBS, was added to each well. Incubation was carried out at room temperature for 2 h (all subsequent incubations were carried out on a shaker at 200 rpm in the dark). After repeated washing, a secondary antibody was added, diluted according to the instructions, and incubated for 2 h at room temperature. Then PBS-T was washed and streptavidin conjugated with horseradish peroxidase (HRP) was added and incubated for 20 min.

TMB substrate (3,3′,5,5′-tetramethylbenzidine) was used for detection. The reaction was stopped by adding 2 M H_2_SO_4_. The optical density was measured at a wavelength of 450 nm with a correction of 540 nm using a microplate photometer. Cytokine concentrations were calculated using calibration curves based on the standards included in the kits.

### 4.11. Assessment of Transendothelial Transport (TET) and Macrophage Migration

TET of LDL and macrophage migration were assessed using a two-chamber Transwell system. The upper chamber (inserts) consisted of a porous insert (BIOFIL, Guangzhou, China) with a pore size of 0.4 μm for TET experiments and 3.0 μm for macrophage migration assays. The lower chamber (wells) corresponded to a well of a 24-well plate (Sarstedt, Germany), in which the insert was placed.

Endothelial cells were seeded onto the membrane of the Transwell inserts at a density of 1 × 10^5^ cells per insert. Cells were cultured in DMEM supplemented with 10% FBS at 37 °C in a humidified atmosphere with 5% CO_2_ for 2–3 days until a confluent monolayer was formed, which was verified visually.

For TET assessment, 500 µL of DMEM containing 100 µg/mL of isolated human LDL was added to the upper chamber. For migration assays, macrophages (1 × 10^5^ cells) were added to the upper chamber. Samples were collected and measurements were performed at 0, 2, 5, and 24 h after the start of the experiment.

Cholesterol concentration in the media from both upper and lower chambers, as well as in cell lysates after extraction, was determined using an enzymatic colorimetric assay (CHOLESTEROL liquicolor kit, HUMAN, Wiesbaden, Germany) based on a modified Folch method. Cholesterol levels were normalized to total protein content measured by the Lowry method. TET rate was defined as the change in cholesterol concentration in the lower chamber.

CD14^+^ monocytes were isolated from peripheral blood by density gradient centrifugation using Ficoll, followed by magnetic separation with nanoparticles (Miltenyi Biotec, Gaithersburg, MD, USA) according to the manufacturer’s protocol. Monocytes were differentiated into macrophages by incubation with granulocyte-macrophage colony-stimulating factor (GM-CSF; 50 ng/mL; Sigma-Aldrich, St. Louis, MO, USA) for 5 days to obtain immature macrophages and further stimulated with lipopolysaccharide (LPS) from Escherichia coli (serotype O26:B6, Sigma-Aldrich, St. Louis, MO, USA) at a concentration of 1 µg/mL for 24 h to induce M1 polarization.

Macrophages (1 × 10^5^ cells) were added to the upper chamber and co-incubated with endothelial monolayers for 2, 5, or 24 h at 37 °C in 5% CO_2_. At each time point, migrated cells in the lower chamber were counted manually in 8 randomly selected fields of view per insert (magnification ×20) using phase-contrast microscopy. Results are expressed as the number of migrated cells per field of view. Experiments were performed in four independent biological replicates.

### 4.12. Sample Preparation

Sample preparation and LC-MS analysis were performed as previously described [4] with minor modifications.

Prior to conditioned medium collection, cell monolayers were washed twice with PBS to remove non-adherent and dead cells, and the culture medium was replaced with serum-free medium supplemented with either LDL (100 µg/mL) or control (without LDL). Conditioned medium was collected after 24 h of incubation. Monolayer integrity was confirmed visually by phase-contrast microscopy prior to each collection. Collected medium was centrifuged at 300× *g* for 5 min to remove cell debris, aliquoted, and stored at −80 °C until analysis. Protein concentration in conditioned medium samples was determined spectrophotometrically using BioSpec-nano (Shimadzu, Kyoto, Japan) prior to sample preparation. Equal protein amounts (10 µg per sample) were used for downstream analysis.

Protein reduction, alkylation, and enzymatic digestion were performed according to a previously described protocol [48] with minor modifications. Briefly, 10 µg of protein sample was mixed with 10 µL of sodium deoxycholate (SDC, Sigma-Aldrich, St. Louis, MO, USA)-based buffer (pH 8.5) containing 100 mM Tris-HCl (Sigma-Aldrich, St. Louis, MO, USA), 1% (*w*/*v*) SDC, 10 mM TCEP (Sigma-Aldrich, St. Louis, MO, USA), and 20 mM chloroacetamide (Sigma-Aldrich, St. Louis, MO, USA).

The mixture was sonicated in a water bath sonicator for 1 min, incubated at 85 °C for 10 min, and then cooled to room temperature. Trypsin (Promega, Madison, WI, USA) dissolved in 100 mM Tris-HCl (pH 8.5) was added at an enzyme-to-protein ratio of 1:50 (*w*/*w*), and samples were incubated overnight at 37 °C for protein digestion.

After digestion, peptides were acidified by adding 50 µL of 2% trifluoroacetic acid (TFA, Sigma-Aldrich, St. Louis, MO, USA) mixed with 50 µL of ethyl acetate (Sigma-Aldrich, St. Louis, MO, USA) and subsequently loaded onto SDB-RPS StageTips containing two 14-gauge disks (Empore, 3M, St. Paul, MN, USA). StageTips were centrifuged at 300× *g* until the solution completely passed through the sorbent (typically ~4 min).

The StageTips were then washed twice with 100 µL of 1% TFA/ethyl acetate (1:1) and once with 50 µL of 0.2% TFA. Peptides were eluted into clean tubes using 60 µL of 60% acetonitrile/5% ammonia solution (Merck, Darmstadt, Germany) by centrifugation at 300× g. The eluates were dried under vacuum and stored at −80 °C until analysis.

Prior to LC–MS analysis, peptides were reconstituted in 20 µL of 2% acetonitrile/0.1% TFA and briefly sonicated for 1 min.

### 4.13. DIA-LC-MS Analysis

Data-independent acquisition liquid chromatography–mass spectrometry (DIA-LC-MS) analysis was performed as described previously [19] with minor modifications. Peptides were loaded onto a home-made trap column (50 × 0.1 mm) packed with Reprosil-Pur 200 C18-AQ resin (5 µm; Dr. Maisch GmbH, Ammerbuch, Germany) using a loading buffer containing 2% acetonitrile (ACN), 98% H_2_O, and 0.1% trifluoroacetic acid (TFA) at a flow rate of 4 µL/min.

Peptide separation was carried out at room temperature on a home-packed fused-silica analytical column (300 × 0.1 mm) filled with Reprosil-Pur C18-AQ resin (1.9 µm; Dr. Maisch). The emitter was prepared using a P2000 laser puller (Sutter Instrument, Novato, CA, USA).

Reversed-phase liquid chromatography was performed using an Ultimate 3000 Nano LC system (Thermo Fisher Scientific, Waltham, MA, USA) coupled to an Orbitrap Tribrid Lumos mass spectrometer (Thermo Fisher Scientific, Waltham, MA, USA) via a nano-electrospray ionization source (Thermo Fisher Scientific, Waltham, MA, USA). Mobile phase A consisted of water containing 0.1% (*v*/*v*) formic acid (FA, Thermo Fisher Scientific, Waltham, MA, USA), and mobile phase B consisted of acetonitrile containing 0.1% FA and 20% (*v*/*v*) water.

Peptides were eluted from the trap column at a flow rate of 500 nL/min using a linear gradient as follows:

3% B for 3 min;

3–6% B for 5 min;

6–40% B for 53 min;

40–60% B for 4 min;

60% B for 2 min;

60–99% B for 0.1 min;

99% B for 2 min;

99–3% B for 0.1 min at a flow rate of 500 nL/min.

Mass spectrometric data were acquired in DIA mode. For overlapping-window DIA-MS acquisition, MS1 spectra were collected in the *m*/*z* range of 495–745 at a resolution of 15,000 with a standard AGC target. MS2 spectra were acquired in the *m*/*z* range of 200–1800 at a resolution of 50,000, with a normalized AGC target of 2000%, maximum injection time set to “auto,” and stepped normalized collision energies of 22, 26, and 30%.

The isolation window width was set to 4 Da.

The complete raw proteomics dataset containing LFQ intensity values for all identified proteins across all samples is provided as Supplementary Table S1.

### 4.14. DIA-NN Data Analysis

Search parameters of DIA-NN [49] (version 2.2.0) were set as follows: precursor FDR 1%; scan window set to 0; isotopologues and MBR turned on; protein inference at gene level; heuristic protein inference enabled; quantification strategy set to Quant UMS (high precision); neural network classifier single-pass mode (cross-validated); mass accuracy at MS1 and MS2 set to both 0. The settings for in silico library generation (Homo sapiens ref. proteome_UP000005640) were as follows: Trypsin/P with maximum 1 missed cleavage; protein N-terminal M excision on; Carbamidomethyl on C as fixed modification; oxidation M and Ac(N-term) as variable modifications; maximum variable modifications 1; peptide length from 7 to 30; precursor charge 1–4; precursor *m*/*z* from 300 to 1800; fragment *m*/*z* from 200 to 1800.

### 4.15. RNA Quality Control and cDNA Library Preparation (Transcriptome Analysis)

RNA concentration was measured using a Qubit 4.0 fluorometer (Thermo Fisher Scientific, Waltham, MA, USA) with the Equalbit RNA HS Assay Kit (Vazyme, Nanjing, China). RNA quality and integrity were assessed using an Agilent 4150 TapeStation system (Agilent Technologies, Santa Clara, CA, USA) with High Sensitivity RNA ScreenTape and RNA ScreenTape kits.

Ribosomal RNA was depleted using the Vazyme Ribo-off rRNA Depletion Kit (Vazyme, China). The resulting RNA samples were immediately used for library preparation with the VAHTS Universal V8 RNA-seq Library Prep Kit for Illumina (Vazyme, China).

The concentration of cDNA libraries was determined fluorometrically using the Qubit 4.0 fluorometer (Thermo Fisher Scientific, Waltham, MA, USA) with the Equalbit 1× dsDNA HS Assay Kit (Vazyme, China). Library quality was evaluated using the Agilent 4150 TapeStation system (Agilent Technologies, Santa Clara, CA, USA) with the High Sensitivity D1000 ScreenTape kit.

Leading edge gene lists for all six analyzed pathways across all four comparisons are provided in Supplementary Table S2.

### 4.16. Statistical Analysis

For PCR and ELISA all experiments were performed using at least six independent biological replicates. For each biological replicate, measurements were carried out in a minimum of two technical replicates. Statistical significance was assessed using the Kruskal–Wallis test and the Conover multiple comparison test. For all results, *p* < 0.05 was considered significant. Statistical data analysis was performed using the Python (version 3.10.4) programming language and the SciPy and scikit-learn libraries.

For mass spectrometry-based secretome and transcriptome analysis we used three independent biological replicates. Before statistical analysis the data were filtered out in order to obtain the list of proteins for the secretome. The methodology was previously published [47] and reproduced by us in previous work [50].

The resulting list of proteins was used for further statistical analysis in Perseus (version 2.0.6.0). Only protein groups with valid maxLFQ values in at least 2 of 3 samples per group were used for quantification. Missing values were imputed from normal distribution with 0.3 intensity distribution sigma width and 2.8 intensity distribution center downshift. Statistical significance of differences between experimental groups was assessed using analysis of variance (ANOVA) with permutation-based FDR 5% followed by correction for multiple comparisons using Tukey’s HSD test. Differences were considered statistically significant at *p* < 0.05.

For the analysis of transendothelial transport (TET) and macrophage migration data, Fisher’s test with Dunn’s correction for multiple comparisons, the Mann–Whitney U test with Bonferroni correction, and the Kruskal–Wallis test followed by Conover’s post hoc test were applied.

Results were described using the median and the 25th and 75th percentiles. Normalization of cholesterol accumulation in the lower and upper chambers was performed relative to the mean value of the control group. For comparison of the cholesterol-to-total protein ratio, min–max normalization was applied, scaling values to a range from 0 to 1.

Prepared cDNA libraries were sequenced on the Genolab M platform (GeneMind, Shenzhen, Guangdong, China) using single-end 75 bp reads.

Sequencing quality control was performed using FastQC (https://www.bioinformatics.babraham.ac.uk/projects/fastqc (accessed on 15 November 2025)), QoRTs [60], and MultiQC [61]. Reads were aligned to the human reference genome using the STAR aligner [62], with the GRCh38 genome assembly and GENCODE v42 annotation.

Gene-level read counts were obtained using featureCounts [63]. Normalization for sequencing depth and differential expression analysis between experimental groups were performed using the DESeq2 package [64] in the R environment.

Gene set enrichment analysis (GSEA) was conducted using the fgsea package (https://www.biorxiv.org/content/10.1101/060012v3 (accessed on 15 November 2025)), based on gene lists ranked by expression levels and *p*-values. The following gene set databases were used: go biological process.

For GSEA, pathways with FDR q-value < 0.25 were considered as showing nominally significant enrichment, consistent with the exploratory nature of the analysis and the recommended threshold for hypothesis-generating studies with limited sample size [19].

Results of differential expression and pathway enrichment analyses were visualized using the ggplot2 (v3.5.1), pheatmap (v1.0.12), and fgsea (v1.28.0) packages in R (v4.4.1).

To select the appropriate statistical test, data normality was assessed using the Shapiro–Wilk test and by visual inspection of quantile–quantile (Q–Q) plots. PCR and ELISA measurements demonstrated deviation from a normal distribution and considerable variability; therefore, the non-parametric Kruskal–Wallis test was applied, as it does not assume normality.

For the mass spectrometry and transcriptome data (n = 3 biological replicates), formal testing did not indicate significant deviation from normality. However, with such a small sample size, reliable determination of the underlying distribution is statistically limited. The data exhibited low within-group variability and comparable variances across groups. Considering the relative robustness of parametric methods to moderate deviations from normality and the limitations of non-parametric tests with extremely small sample sizes, we used ANOVA for the analysis of mass spectrometry and transcriptome data.

For flow cytometry data, differences in FSC-A and SSC-A parameters between TECs and MVECs were assessed using Student’s independent samples *t*-test (n = 5 biological replicates per group). Data are presented as mean ± standard deviation. Normal distribution of the data was confirmed using the Shapiro–Wilk test prior to parametric testing.

Heatmap visualization of secretome proteomic data: raw label-free quantification (LFQ) intensities were log_2_-transformed prior to analysis. For each protein, z-score normalization was applied row-wise across all samples using only detected (non-zero) values, according to the formula: z = (x − μ)/σ, where x is the log_2_ LFQ value of an individual replicate, μ is the mean, and σ is the standard deviation calculated across all detected replicates of a given protein. This approach centers each protein’s expression profile around its own mean and scales it by its variability, enabling visual comparison of relative abundance patterns across groups regardless of differences in absolute signal intensity between proteins. Samples with LFQ = 0 in all replicates of a given group were classified as not detected (ND) and excluded from z-score calculation; these samples are displayed as grey cells in the heatmap. The heatmap was generated using a diverging Red–Blue color scale (RdBu_r), where red indicates above-average abundance and blue indicates below-average abundance relative to the protein-specific mean. Groups are separated by vertical dividers, with individual biological replicates (n = 3 per group) shown as separate columns.

Visualization of all results was carried out using standard Python libraries: Seaborn (version 0.13.2), Matplotlib (version 3.8.4).

## Data Availability

The original contributions presented in this study are included in the article. Further inquiries can be directed to the corresponding author.

## Supplementary Materials

The following supporting information can be downloaded at: https://www.mdpi.com/article/10.3390/ijms27135728/s1.

## Abbreviations

The following abbreviations are used in this manuscript:

ECs	Endothelial cells
ED	Endothelial dysfunction
TECs	Typical endothelial cells
MVECs	Multinucleated variant endothelial cells
HUVEC	Human umbilical vein endothelial cells
LDL	Low-density lipoprotein
oxLDL	Oxidized low-density lipoprotein
TET	Transendothelial transport
ECM	Extracellular matrix
GSEA	Gene set enrichment analysis
NES	Normalized enrichment score
FDR	False discovery rate
LFQ	Label-free quantification
DIA-LC-MS	Data-independent acquisition liquid chromatography–mass spectrometry
qPCR	Quantitative polymerase chain reaction
ELISA	Enzyme-linked immunosorbent assay
IL	Interleukin
IL-6	Interleukin-6
IL-8	Interleukin-8
TNF	Tumor necrosis factor
NF-κB	Nuclear factor kappa B
VWF	von Willebrand factor
CDH5	VE-cadherin
TGFB1	Transforming growth factor beta 1
MMP2	Matrix metalloproteinase-2
DCN	Decorin
LAMC2	Laminin subunit gamma-2
eNOS	Endothelial nitric oxide synthase
NO	Nitric oxide
PEG	Polyethylene glycol
GM-CSF	Granulocyte-macrophage colony-stimulating factor
LPS	Lipopolysaccharide
PBS	Phosphate-buffered saline
PBS-T	Phosphate-buffered saline with Tween-20
BSA	Bovine serum albumin
FBS	Fetal bovine serum
ANOVA	Analysis of variance
SD	Standard deviation
SEM	Standard error of the mean
TFA	Trifluoroacetic acid
ACN	Acetonitrile
TMB	3,3′,5,5′-Tetramethylbenzidine
FSC	Forward scatter
SSC	Side scatter
PFA	Paraformaldehyde
DAPI	4′,6-diamidino-2-phenylindole

## References

[1] Gimbrone M.A., García-Cardeña G. (2016). Endothelial Cell Dysfunction and the Pathobiology of Atherosclerosis. Circ. Res..

[2] Tokunaga O., Fan J., Watanabe T. (1989). Atherosclerosis- and age-related multinucleated variant endothelial cells in primary culture from human aorta. Am. J. Pathol..

[3] Zuckerman J.E., Brealey J., Yabu J.M., Chang A. (2022). Large Multinucleated Variant Endothelial Cells in Allograft Kidney Microvasculature: A Biopsy Series. Kidney Med..

[4] Cherednichenko V., Kiseleva D., Khovantseva U., Breshenkov D., Ziganshin R., Dymova O., Kirichenko T., Charchyan E., Markin A.M. (2026). Functional Differences Between Typical and Multinucleated Endothelial Cells Under Low-Density Lipoprotein Exposure. Int. J. Mol. Sci..

[5] Tokunaga O., Satoh T., Yamasaki F., Wu L. (1998). Multinucleated variant endothelial cells (MVECs) in human aorta: Chromosomal aneuploidy and elevated uptake of LDL. Semin. Thromb. Hemost..

[6] Watanabet T., Tokunagao O. (1990). Multinucleated variant endothelial cell. Its characterization and relation to atherosclerosis. Ann. N. Y. Acad. Sci..

[7] Wu L., Satoh T., Tokunaga O. (1999). Formation of multinucleated variant endothelial cells in vitro and investigation of MVECs’ features. Fukuoka Igaku Zasshi.

[8] Zhou X., Zhou M., Zheng M., Tian S., Yang X., Ning Y., Li Y., Zhang S. (2022). Polyploid giant cancer cells and cancer progression. Front. Cell Dev. Biol..

[9] Yang J., Shen M.H. (2006). Polyethylene Glycol-Mediated Cell Fusion. Methods Mol. Biol..

[10] Ishida T., Li W., Liu Z., Kiwada H. (2006). Stimulatory effect of polyethylene glycol (PEG) on gene expression in mouse liver following hydrodynamics-based transfection. J. Gene Med..

[11] Aird W.C. (2007). Phenotypic heterogeneity of the endothelium: I. Structure, function, and mechanisms. Circ. Res..

[12] Libby P., Buring J.E., Badimon L., Hansson G.K., Deanfield J., Bittencourt M.S., Tokgözoğlu L., Lewis E.F. (2019). Atherosclerosis. Nat. Rev. Dis. Primers.

[13] Tabas I., Williams K.J., Borén J. (2007). Subendothelial lipoprotein retention as the initiating process in atherosclerosis: Update and therapeutic implications. Circulation.

[14] Lusis A.J. (2000). Atherosclerosis. Nature.

[15] Ley K., Laudanna C., Cybulsky M.I., Nourshargh S. (2007). Getting to the site of inflammation: The leukocyte adhesion cascade updated. Nat. Rev. Immunol..

[16] Krüger-Genge A., Blocki A., Franke R.P., Jung F. (2019). Vascular Endothelial Cell Biology: An Update. Int. J. Mol. Sci..

[17] Hu W.N., Duan Z.Y., Wang Q., Zhou D.H. (2019). The suppression of ox-LDL-induced inflammatory response and apoptosis of HUVEC by lncRNA XIAT knockdown via regulating miR-30c-5p/PTEN axis. Eur. Rev. Med. Pharmacol. Sci..

[18] Xia F., Zeng Q. (2022). miR-125a-3p aggravates ox-LDL-induced HUVEC injury through BAMBI. J. Biochem. Mol. Toxicol..

[19] Kawashima Y., Nagai H., Konno R., Ishikawa M., Nakajima D., Sato H., Nakamura R., Furuyashiki T., Ohara O. (2022). Single-Shot 10K Proteome Approach: Over 10,000 Protein Identifications by Data-Independent Acquisition-Based Single-Shot Proteomics with Ion Mobility Spectrometry. J. Proteome Res..

[20] Subramanian A., Tamayo P., Mootha V.K., Mukherjee S., Ebert B.L., Gillette M.A., Paulovich A., Pomeroy S.L., Golub T.R., Lander E.S. (2005). Gene set enrichment analysis: A knowledge-based approach for interpreting genome-wide expression profiles. Proc. Natl. Acad. Sci. USA.

[21] Pollard T.D., Cooper J.A. (2009). Actin, a Central Player in Cell Shape and Movement. Science.

[22] Lip G.Y.H., Blann A. (1997). von Willebrand factor: A marker of endothelial dysfunction in vascular disorders?. Cardiovasc. Res..

[23] Soeki T., Tamura Y., Shinohara H., Sakabe K., Onose Y., Fukuda N. (2004). Elevated concentration of soluble vascular endothelial cadherin is associated with coronary atherosclerosis. Circ. J..

[24] Galis Z.S., Khatri J.J. (2002). Matrix Metalloproteinases in Vascular Remodeling and Atherogenesis. Circ. Res..

[25] Pardali E., Ten Dijke P. (2012). TGFβ Signaling and Cardiovascular Diseases. Int. J. Biol. Sci..

[26] Pober J.S., Sessa W.C. (2007). Evolving functions of endothelial cells in inflammation. Nat. Rev. Immunol..

[27] Liu Y., Li Y., Chen M., Liu Y., Liang J., Zhang Y., Qian Z.J. (2022). Mechanism of two alkaloids isolated from coral endophytic fungus for suppressing angiogenesis in atherosclerotic plaque in HUVEC. Int. Immunopharmacol..

[28] Zheng H., Pei Y., Zhou C., Hong P., Qian Z.J. (2023). Amelioration of atherosclerosis in ox-LDL induced HUVEC by sulfated polysaccharides from Gelidium crinale with antihypertensive activity. Int. J. Biol. Macromol..

[29] Wu X., Zheng X., Cheng J., Zhang K., Ma C. (2020). LncRNA TUG1 regulates proliferation and apoptosis by regulating miR-148b/IGF2 axis in ox-LDL-stimulated VSMC and HUVEC. Life Sci..

[30] Stenmark H., Olkkonen V.M. (2001). The Rab GTPase family. Genome Biol..

[31] Spano A., Sciola L. (2023). Polyploid cell dynamics and death before and after PEG-treatment of a NIH/3T3 derived culture: Vinblastine effects on the regulation of cell subpopulations heterogeneity. Cell Div..

[32] Weiss S.N., Legato J.M., Liu Y., Vaccaro C.N., Da Silva R.P., Miskiel S., Gilbert G.V., Hakonarson H., Fuller D.A., Buono R.J. (2024). An analysis of differential gene expression in peripheral nerve and muscle utilizing RNA sequencing after polyethylene glycol nerve fusion in a rat sciatic nerve injury model. PLoS ONE.

[33] Turner M.D., Nedjai B., Hurst T., Pennington D.J. (2014). Cytokines and chemokines: At the crossroads of cell signalling and inflammatory disease. Biochim. Biophys. Acta (BBA)-Mol. Cell Res..

[34] Hunter C.A., Jones S.A. (2015). IL-6 as a keystone cytokine in health and disease. Nat. Immunol..

[35] Cotran R.S., Pober J.S. (1990). Cytokine-endothelial interactions in inflammation, immunity, and vascular injury. J. Am. Soc. Nephrol..

[36] Blokhina T., Kirichenko T., Markina Y., Khovantseva U., Melnikov I., Guseva O., Bazanovich S., Kozlov S., Orekhov A. (2025). Features of the monocyte inflammatory response in patients with premature coronary artery disease. Biophys. Rep..

[37] Watson C., Whittaker S., Smith N., Vora A.J., Dumonde D.C., Brown K.A. (1996). IL-6 acts on endothelial cells to preferentially increase their adherence for lymphocytes. Clin. Exp. Immunol..

[38] Harada A., Sekido N., Akahoshi T., Wada T., Mukaida N., Matsushima K. (1994). Essential involvement of interleukin-8 (IL-8) in acute inflammation. J. Leukoc. Biol..

[39] Khovantseva U., Kiseleva D., Cherednichenko V., Breshenkov D., Matveeva D., Kirichenko T., Markina Y., Charchyan E., Markin A. (2026). Integrative Analysis of VSMC, Macrophage, and Fibroblast Responses to LDLs in Aortic Pathologies. Int. J. Mol. Sci..

[40] Yu H., Huang X., Ma Y., Gao M., Wang O., Gao T., Shen Y., Liu X. (2013). Interleukin-8 Regulates Endothelial Permeability by Down-regulation of Tight Junction but not Dependent on Integrins Induced Focal Adhesions. Int. J. Biol. Sci..

[41] Li A., Dubey S., Varney M.L., Dave B.J., Singh R.K. (2003). IL-8 Directly Enhanced Endothelial Cell Survival, Proliferation, and Matrix Metalloproteinases Production and Regulated Angiogenesis. J. Immunol..

[42] Tokunaga O., Satoh T., Yu S. (2002). Multinucleated variant endothelial cells (MVECs) have a greater capacity for LDL cholesterol uptake than typical mononuclear endothelial cells (TECs). J. Atheroscler. Thromb..

[43] Theodorou K., Boon R.A. (2018). Endothelial Cell Metabolism in Atherosclerosis. Front. Cell Dev. Biol..

[44] Giráldez A.P., Gómez A.B., Calmarza P., Bailo P.S., Khialani A.D., Breva S.M., Sainz-Pastor N., Fort Gallifa I. (2026). Oxidative Stress and Its Role in Vascular Damage and Atherosclerosis. Int. J. Mol. Sci..

[45] Khatana C., Saini N.K., Chakrabarti S., Saini V., Sharma A., Saini R.V., Saini A.K. (2020). Mechanistic Insights into the Oxidized Low-Density Lipoprotein-Induced Atherosclerosis. Oxid. Med. Cell Longev..

[46] Soccio R.E., Breslow J.L. (2004). Intracellular cholesterol transport. Arter. Thromb. Vasc. Biol..

[47] Maxfield F.R., van Meer G. (2010). Cholesterol, the central lipid of mammalian cells. Curr. Opin. Cell Biol..

[48] Wilhelm L.P., Wendling C., Védie B., Kobayashi T., Chenard M., Tomasetto C., Drin G., Alpy F. (2017). STARD3 mediates endoplasmic reticulum-to-endosome cholesterol transport at membrane contact sites. EMBO J..

[49] Shen Q., Wu M.H., Yuan S.Y. (2009). Endothelial contractile cytoskeleton and microvascular permeability. Cell Health Cytoskelet..

[50] Aman J., Margadant C. (2023). Integrin-Dependent Cell-Matrix Adhesion in Endothelial Health and Disease. Circ. Res..

[51] Davis G.E., Senger D.R. (2005). Endothelial extracellular matrix: Biosynthesis, remodeling, and functions during vascular morphogenesis and neovessel stabilization. Circ. Res..

[52] Khovantseva U., Kiseleva D., Cherednichenko V., Chakal D., Breshenkov D., Markina Y., Ziganshin R., Charchyan E., Markin A. (2025). The new perspective on understanding the mechanisms of cardiovascular diseases development. Sci. Rep..

[53] Iozzo R.V., Schaefer L. (2015). Proteoglycan form and function: A comprehensive nomenclature of proteoglycans. Matrix Biol..

[54] Khovantseva U., Markina Y., Kirichenko T., Goncharova K., Kiseleva D., Cherednichenko V., Markin A. (2025). Phenotypic Switching of VSMCs in the Development of CVDs: Focus on miRs. Int. J. Mol. Sci..

[55] Khovantseva U.S., Kiseleva D.G., Cherednichenko V.R., Fotin D.P., Bogatyreva A.I., Boyarskaya N.V., Chakal D.A., Breshenkov D.G., Markina Y.V., Malashicheva A.B. (2024). Functional features of smooth muscle cells of the human aortic wall and their role in the pathogenesis of aneurysms. Morphology.

[56] Kiseleva D.G., Ziganshin R.K., Fotin D.P., Markin A.M. (2024). Proatherogenic proteomic profile of LDL isolated from plasma of patients with diabetes mellitus: Immunological aspects. Russ. J. Immunol..

[57] Kiseleva D., Kolmogorov V., Cherednichenko V., Khovantseva U., Bogatyreva A., Markina Y., Gorelkin P., Erofeev A., Markin A. (2024). Effect of LDL Extracted from Human Plasma on Membrane Stiffness in Living Endothelial Cells and Macrophages via Scanning Ion Conductance Microscopy. Cells.

[58] Khovantseva U.S., Matveeva D.K., Chakal D.A., Breshenkov D.G., Charchyan E.R. (2024). Phagocytic activity and proinflammatory activation potential of smooth muscle cells of the Tunica intima of human aorta under experimental conditions. Russ. J. Immunol..

[59] Bogachkov Y.Y., Chen L., Le Master E., Fancher I.S., Zhao Y., Aguilar V., Oh M.J., Wary K.K., DiPietro L.A., Levitan I. (2020). LDL induces cholesterol loading and inhibits endothelial proliferation and angiogenesis in Matrigels: Correlation with impaired angiogenesis during wound healing. Am. J. Physiol. Cell Physiol..

[60] Hartley S.W., Mullikin J.C. (2015). QoRTs: A comprehensive toolset for quality control and data processing of RNA-Seq experiments. BMC Bioinform..

[61] Ewels P., Magnusson M., Lundin S., Käller M. (2016). MultiQC: Summarize analysis results for multiple tools and samples in a single report. Bioinformatics.

[62] Dobin A., Davis C.A., Schlesinger F., Drenkow J., Zaleski C., Jha S., Batut P., Chaisson M., Gingeras T.R. (2013). STAR: Ultrafast universal RNA-seq aligner. Bioinformatics.

[63] Liao Y., Smyth G.K., Shi W. (2014). featureCounts: An efficient general purpose program for assigning sequence reads to genomic features. Bioinformatics.

[64] Love M.I., Huber W., Anders S. (2014). Moderated estimation of fold change and dispersion for RNA-seq data with DESeq2. Genome Biol..

