# Supplementary figures and images for: Lipid-Induced Endothelial Dysfunction: Pro-Atherogenic Properties of Multinucleated Variant Endothelial Cells
Source: Int J Mol Sci. 2026 Jun 25;27(13):5728. doi: 10.3390/ijms27135728 (PMC13362034; doi:10.3390/ijms27135728)

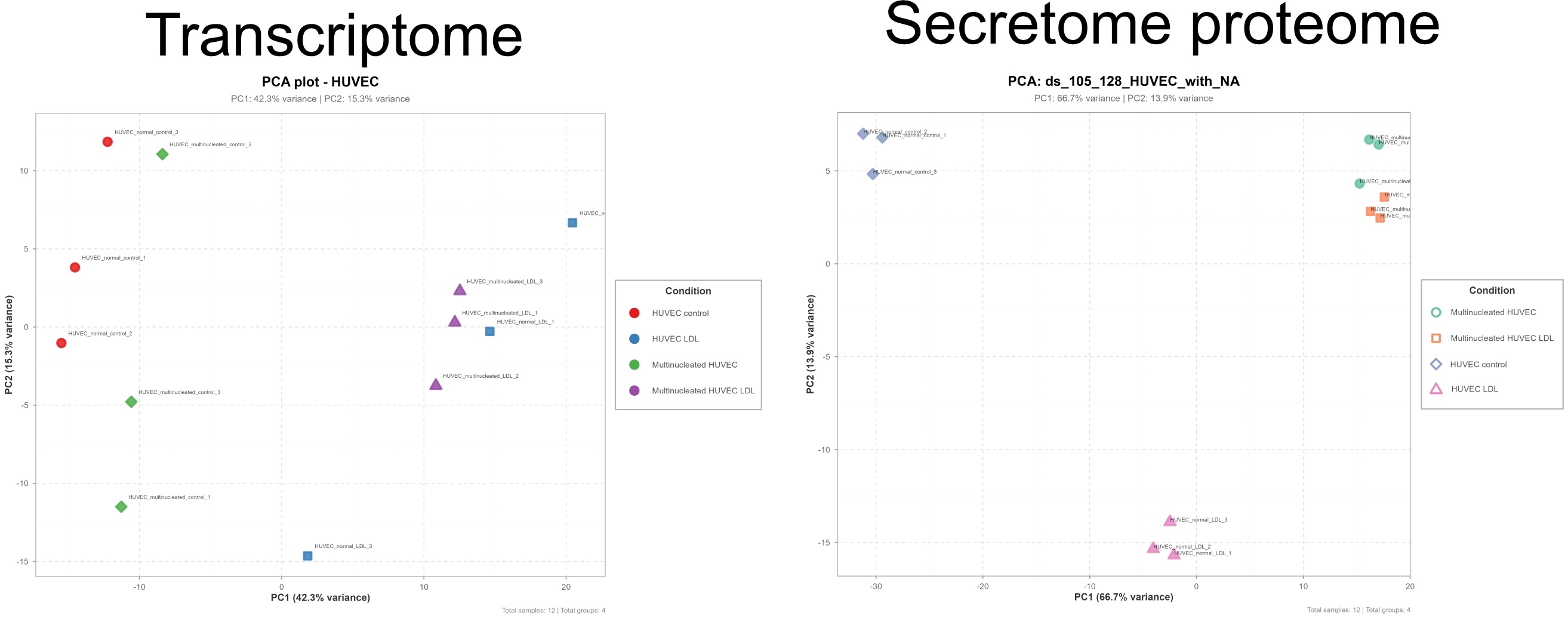

Supplement: Supplementary file 1 [file ijms-27-05728-s001.zip › Figure S1.jpg]
